# Importance of glycosylation in the interaction of Tamm‐Horsfall protein with collectin‐11 and acute kidney injury

**DOI:** 10.1111/jcmm.15046

**Published:** 2020-02-11

**Authors:** Kunjing Gong, Min Xia, Yaqin Wang, Lufeng Bai, Wantao Ying, Fengxue Zhu, Yuqing Chen

**Affiliations:** ^1^ Renal Division Department of Medicine Peking University First Hospital Beijing China; ^2^ Institute of Nephrology Peking University Beijing China; ^3^ Key Laboratory of Renal Disease Ministry of Health of China Beijing China; ^4^ Key Laboratory of Chronic Kidney Disease Prevention and Treatment Ministry of Education Beijing China; ^5^ State Key Laboratory of Proteomics Beijing Proteome Research Center National Center for protein science (Beijing) Beijing Institute of lifeomics Beijing China; ^6^ Department of Critical Care Medicine Peking University People's Hospital Beijing China

**Keywords:** acute kidney injury, collectin‐11, glycosylation, lectin pathway, Tamm‐Horsfall protein

## Abstract

Both Tamm‐Horsfall protein (THP) and collectin‐11 (CL‐11) are important molecules in acute kidney injury (AKI). In this study, we measured the change of glycosylation of THP in patients with AKI after surgery, using MALDI‐TOF MS and lectin array analysis. The amount of high‐mannose and core fucosylation in patients with AKI were higher than those in healthy controls. In vitro study showed that THP could bind to CL‐11 with affinity at 9.41 × 10^−7^ mol/L and inhibited activation of complement lectin pathway. The binding affinity decreased after removal of glycans on THP. Removal of fucose completely ablated the binding between the two proteins. While removal of high‐mannose or part of the N‐glycan decreased the binding ability to 30% or 60%. The results indicated that increase of fucose on THP played an important role via complement lectin pathway in AKI.

## 1 INTRODUCTION

Tamm‐Horsfall protein (THP) is the most abundant protein in normal human urine, and carbohydrate forms 25% of the molecular mass of THP.^1^ THP is considered as an important protective factor during acute kidney injury (AKI).^2^ Lower level of urinary THP associates with increased risk of AKI in patients receiving cardiac surgery.^3^ The function of THP in AKI remains unclear and the immunological activities of THP are gradually getting noticed.^4^ One of the immunomodulation functions of THP is its binding capacity with complement system.^4^ Previous studies revealed that Tamm‐Horsfall protein could bind to complement 1q (C1q) with high affinity.^5^, ^6^ Our work also identified that THP could bind to complement factor H (cFH), and enhance the cofactor activity of cFH in degradation of C3b.^7^ The interactions between THP and complements suggest that THP may be a regulating protein in complement activation. Meanwhile, complement activation gradually presents an important role in the progression of various forms of renal disease 8, 9 and may also involve in AKI.^10^, ^11^

Recently, collectin‐11 (CL‐11), an initial molecule of lectin pathway in complement activation,^12^ is identified to be involved in AKI and chronic renal inflammation.^13^, ^14^ CL‐11 engages with L‐fucose during hypoxia‐ or hypothermia stress, activates the complement lectin pathway and induces renal epithelial cell injury.^13^ CL‐11 can also promote chronic renal inflammation and tubulointerstitial fibrosis through its effects on leukocyte chemotaxis and renal fibroblast proliferation.^14^ As we mentioned above, THP can bind with C1q and cFH and participate in the regulation of classic and alternative complement pathways. Thus, it is interesting to know whether THP can also influence lectin pathway.

Collectin‐11 activates lectin pathway via binding with fucose and mannose.^15^ Removal of carbohydrate from THP reduced the ability of THP binding of C1q and eliminated the ability of THP to protect against complement activation.^16^ Meanwhile, abnormal glycosylation of THP was observed in patients with interstitial cystitis as well as in patients accepting renal transplantation,^17^, ^18^ and the disturbance of glycosylation influenced immunoreaction of THP, indicated that glycans on THP mediated the pathogenic process of urologic diseases.^18^, ^19^

Thus, our hypothesis is that THP can bind to CL‐11 and influence complement activation. The interaction is mediated by glycans on THP and change of glycosylation on THP occurred during kidney stress. We first measured the binding between THP and CL‐11 and then explored whether the binding between the two molecules influenced the activation of complement system. Finally, we investigated the change of glycosylation of THP during kidney stress.

## 2 MATERIALS AND METHODS

### 2.1 Isolation of THP

We purified THP from urine using the rapid isolation method.^20^ Briefly, urine was first filtered through diatomaceous earth in Buchner funnel; then, the earth was washed by PBS and dissolved in deionized water. The mixture was centrifuged at 20 000 *g* and the pellet was discarded. Sodium phosphate buffer containing 0.14 mol/L NaCl was added into the suspension. The previous steps were repeated to get the suspension again. The suspension was dialysed and the protein was concentrated with ultrafiltration cartridge (30‐kD MW cut‐off, Millipore, USA). Purity of THP was measured by 10% tSDS‐PAGE with Coomassie brilliant blue staining. Purified protein samples were lyophilized and stored at −80°C for future experiments.

### 2.2 Binding of THP with CL‐11 detected by microscale thermophoresis (MST)

Binding capacity of THP and CL‐11 was detected by MST (Monolith NT.115, NanoTemper, Germany). THP was labelled with red fluorescent using Monolith NT™ Protein Labeling Kit. The MST power was set at 40%, and LED excitation power was set at 20%. THP labelled with fluorescent was kept at 180 nM. CL‐11 was diluted from the concentration of 9 μmol/L. Measurement of the binding affinity was performed in standard treated capillaries. The data were analysed with MO Affinity Analysis software, and the binding affinity was calculated using the Hill model.

### 2.3 Binding of THP with CL‐11, Mannose‐binding lectin (MBL) and ficolins detected by enzyme‐linked immunosorbent assay (ELISA)

Binding of THP with CL‐11, MBL, and ficolins was detected by ELISA. The microplates were first coated with 4μg/ml recombinant CL‐11, MBL or ficolin1, 2, −3 (R&D) in 0.1 mol/L carbonate buffer (pH 9.6) at 4°C for overnight. The plates were blocked with 1% BSA/PBS at 37°C for 1 hour, and THP was added with concentration varied from 0.3125 to10 μg/mL at 37°C for 1 hour. For CL‐11 and ficolins binding assay, THP was diluted in VBS buffer with 0.5 mmol/L MgCl_2_ 0.15 mmol/L CaCl_2_. The CaCl_2_ concentration was 1 mmol/L for MBL binding assay. Subsequently, wells were incubated with rabbit anti‐human uromodulin polyclonal antibody (Biomedical Technologies Inc, USA) and mouse anti‐rabbit IgG alkaline phosphatase (Sigma‐Aldrich, USA) both at 37°C for 1 hour. The OD at 405 nm was measured using a microplate reader (iMark, BIO‐RAD).

### 2.4 Removal of N‐glycans of THP

PNGaseF, endoglycosidase H (Endo H) and fucosidase were used to treat the native THP according to the product protocol. PNGaseF is an amidase which removes almost all N‐linked oligosaccharides from glycoproteins. Endo H is a recombinant glycosidase which recognizes the high‐mannose structure of N‐glycosylation. Fucosidase is an exoglycosidase specifically recognizing fucose. N‐glycan was removed both under denatured condition and native condition. In this study, denatured THP (in 100°C) lost the activity of binding to CL‐11; thus, we used the recommended non‐denaturing protocol to digest the THP. ELISA protocol detecting binding capacity of deglycosylated THP with CL‐11 was similar as above.

### 2.5 Haemolytic assay

Haemolytic assay was modified according to the previous study.^21^ In this test, we detected the haemolysis percentage of chicken erythrocytes in the serum with anti‐C1q antibody to exclude the interference of classical complement pathway.^21^ MBL deficient serum (0.03 μg/mL) from a healthy female volunteer was used as the reagent to provide all complement components except MBL. The reaction mixture was 10 mL VBS containing 3 × 10^7^ *S cerevisiae*, 10^9^ chicken erythrocytes and 76 μL anti‐C1q antibody (50 μg/mL Dako). Five microlitre MBL deficient serum diluted in 45 μL THP (concentration: 0, 0.3125, 0.625, 1.25, 2.5, 5, 10 μmol/L) dissolved in VBS (0.5 mmol/L MgCl_2_ 0.15 mmol/L CaCl_2_) and then incubated them at 37°C for 30 minutes. After the incubation, 50 μL serum dilutions containing THP and 100 μL reaction mixture were put on a water‐based incubator operating at 37°C. Subsequently, the erythrocytes were spun down at 2000 g for 10 minutes. Fifty microlitre supernatant in 150 μL water was pipetted in a microplate. Haemolytic activity was measured using a microreader (OD of 405 nm). One hundred percent positive control was erythrocytes in diluted serum without protein sample (THP 0 μmol/L), and negative control was erythrocytes in VBS buffer. BSA dissolved in VBS was a control for THP influence on lectin pathway activation.

### 2.6 Study participants

Twenty patients in intensive care unit (ICU) were recruited in this study. The 20 individuals were admitted into ICU because of various type of surgery, and they were diagnosed as acute kidney injury on an increase in serum creatinine ≥0.3 mg/dL (26.5 μmol/L) within 48 hours or urine volume <0.5 mL/kg/h for 6 hours, based on KDIGO recommendation.^22^ Their characteristics were listed in Table 1. Informed consents were obtained from participants. Urine of all the participants was collected, and Tamm‐Horsfall protein was purified and prepared for further detection of glycans by matrix‐assisted laser desorption/ionization‐time of flight mass spectrometer (MALDI‐TOF MS) and lectin array analysis. We also purified THP from 10 individuals with normal renal function as controls in the analysis.

**Table 1:** Characteristics of the participants

Characteristics	All patients	Patients for MS analysis only	Patients for lectin array only	Patients for both lectin array and MS
Number	20	5	10	5
Male/Female	9/11	2/3	5/5	2/3
Age	61.2 ± 3.3	59.4 ± 6.4	57.0 ± 4.3	71.4 ± 7.1
Creatinine (μmol/L)	146.9 ± 20.8	179.6 ± 38.3	155.4 ± 35.3	97.4 ± 15.4
BUN (mmol/L)	14.2 ± 2.0	17.2 ± 2.8	13.0 ± 2.9	13.7 ± 5.5
Uric acid (μmol/L)	300.6 ± 36.6	398.0 ± 66.9	256.2 ± 56.1	290.0 ± 38.6
AKI Diagnosis criteria				
Increased creatinine	12	2	7	3
Decreased urine output	8	3	3	2
Stage of AKI				
Stage 1	18	4	10	4
Stage 2	2	1	0	1
Stage 3	0	0	0	0

Note

The characteristics of patients were listed above. The patients were diagnosed with AKI based on (1) an increase in serum creatinine ≥ 0.3 mg/dL (26.5 μmol/L) within 48 h or (2) urine volume <0.5 mL/kg/h for 6 h, from KDIGO recommendation. The stage of AKI was also identified according to KDIGO recommendation.^22^ Data were expressed as mean ± SEM.

Abbreviations: AKI, acute kidney injury; BUN, blood urea nitrogen.

### 2.7 Measurement of N‐glycan profile by MALDI‐TOF MS

One hundred microgram THP was used in this experiment. N‐glycan of THP was released by PNGase F (Sigma) after reduction and alkylation. THP was first denatured at 100°C for 10 minutes. And then the protein was reduced in 20 mmol/L dithiothreitol at 56°C for 45 minutes, followed by alkylated with iodoacetamide (50 mmol/L) at room temperature for 1 hour in the dark. De‐glycosylation of the protein was performed in the ammonium bicarbonate (50 mmol/L) buffer at 37°C for 2 hours with 10 μL PNGase F. The mixture was centrifuged with ultrafiltration cartridge (30 kD MW) at 14 500 *g*, 15 minutes to collect the N‐glycans released from THP. Enrichment of the N‐Glycans was performed with HILIC as previous described.^23^ To get an optimized mass spectrum profile, we removed the sialic acid in 0.22% trifluoroacetic acid at 82°C for 2 hours before lyophilized the glycans. The glycans were dissolved with ultrapure water and mixed with matrix by 1:1 ratio. N‐glycan profile was measured with MALDI‐TOF MS (ultrafleXtreme TOF/TOF; Bruker Daltonics). Data were analysed using flexAnalysis (version 3.3).

### 2.8 Lectin microarray screening of purified THP

Lectin array of purified THP was performed using a commercial lectin array kit (GA‐Lectin‐70, Raybiotech, USA). Briefly, 30 μg lyophilized THP was resuspended in ultrapure water. The samples were labelled with biotin, then dialysed overnight at 4°C. The glass slide was blocked with 100 μL dilutions for 30 minutes. Diluted samples were added in testing wells and incubated the array overnight at 4°C. Samples were decanted from each well, and washed the well and glass slide with 1× Wash Buffer I at room temperature with gentle shaking. Subsequently, 80 μL Cy3‐Streptavidin was pipetted into each well. The array plate was incubated at room temperature for 1 hour in the dark. Cy3‐Streptavidin was decanted then washed the wells and glass slide. The glass slide was dried by centrifuging at 480 *g*. for 3 minutes without cap. Finally, the glass slide was scanned at 532 nm, 100% Power with Agilent SureScan Dx Microarray Scanner (Agilent, USA). Previous studies indicated that same carbohydrates on different proteins or tissues cannot be identified by all candidate lectins.^24^, ^25^ Most of the commonly used lectins were included in the array chip, so that we can find more suitable lectins to detect THP glycosylation. This array contains 70 lectins, in order to detect eight specific carbohydrates, including mannose (Man), fucose (Fuc), sialic acid, galactose (Gal), glucose (Glc), lactose (Lac), N‐acetylglactosamine (GalNAC) and N‐acetylglucosamine (GlcNAc).

### 2.9 Statistical analyses

All data were analysed using SPSS 22.0 (IBM, USA). Continuous variables were presented as mean ± SEM. MALDI‐TOF MS data were processed with flexAnalysis (v3.3), and the output intensity list was normalized. *t* Test was used to compare the normalized MS data between AKI patients and healthy controls. Lectin array test was compared using *t* test. False discovery rate (FDR) was used to correct the *P*‐values for multiple testing. Data were considered statistically significant when *P* < .05.

## 3 RESULTS

### 3.1 THP bound with collectin‐11 in a dose‐dependent manner

**Figure 1:**
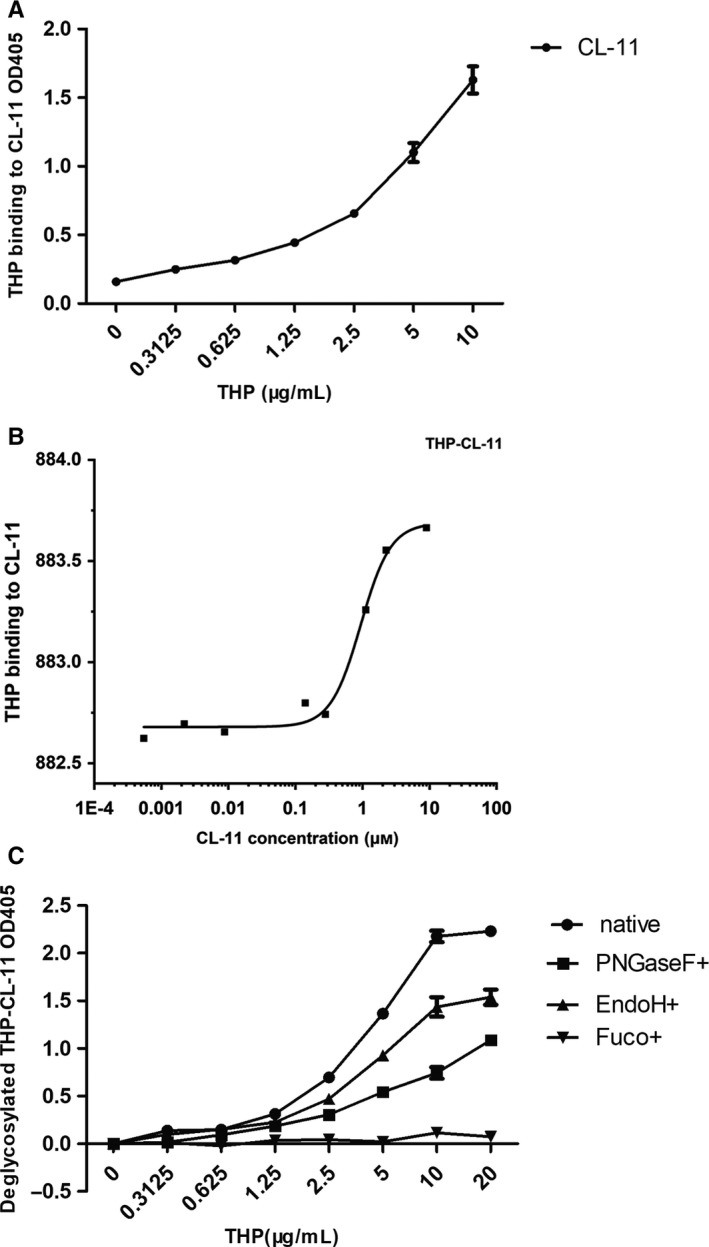
Binding of CL‐11 and THP. A, Binding of CL‐11 and native THP by ELISA. The binding experiment was performed with fixed CL‐11 dose and various THP dose. The experiment was performed in duplicate wells and repeated at least three times. The binding of THP with CL‐11 was presented as value of OD405. Data were expressed as mean ± SEM. B, Binding of CL‐11 and native THP by MST. Interaction of THP with CL‐11 was detected with MST. CL‐11 was diluted into different concentration to bind with THP. The dose‐response curve was fitted using Hill model in the MO. Affinity Analysis Software binding affinity was 9.41 × 10^−7^ mol/L. C, Binding of CL‐11 and deglycosylated THP by ELISA. Glycans of THP were removed by different enzymes. The binding experiment was performed with fixed CL‐11 dose and different dose of deglycosylated THP. The experiment was performed in duplicate wells and repeated at least three times. The binding of deglycosylated THP with CL‐11 was presented as value of OD405. Data points were mean ± SEM of three repeated experiments

The purity of collected Tamm‐Horsfall protein was measured by Coomasie blue stain (Figure S1). The binding capacity of THP with CL‐11 was detected by ELISA and MST. The result showed that THP bound to CL‐11 in a dose‐dependent manner (Figure 1A). The binding capacity was re‐confirmed by MST, with a binding affinity at 9.41 × 10^−7^ mol/L (Figure 1B).

### 3.2 Glycans on THP mediated binding between THP and collectin‐11

PNGaseF was used to remove the carbohydrate side chains in non‐reduced condition. Fucosidase and EndoH were used to remove fucose and high‐mannose glycans from THP. Binding capacity with CL‐11 was measured and compared between native THP and deglycosylated THP. The binding strength between THP and CL‐11 decreased after removal of glycans. Removal of fucose completely ablated the binding between the two proteins. While removal of high‐mannose or part of the N‐glycan decreased the binding ability to 30% or 60% (Figure 1C).

### 3.3 THP inhibited erythrocytes haemolysis mediated by lectin pathway

**Figure 2:**
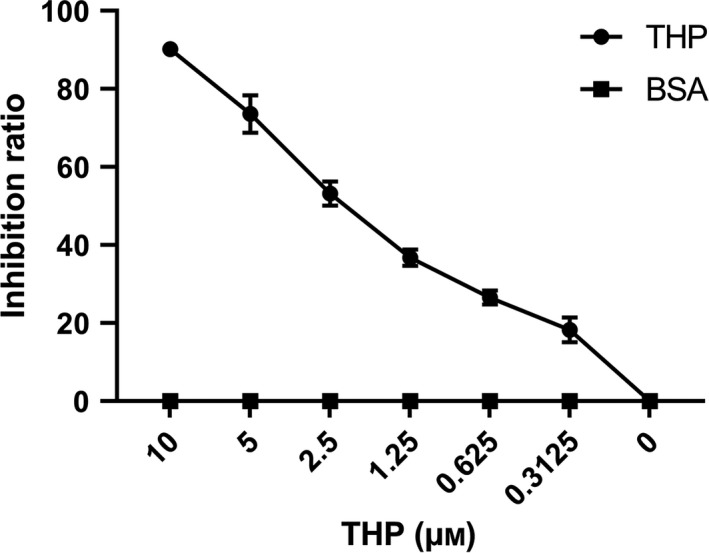
Tamm‐Horsfall protein (THP) inhibited complement lectin pathway activation with haemolysis assay. Lectin pathway activity was detected with a haemolytic assay. Different concentration of THP was added. And BSA at different level was also added as control to exclude influence of adding protein. The effect of THP on haemolysis was presented as inhibition ratio. Higher inhibition ratio meant more inhibition of haemolysis. Inhibition ratio was calculated as formula below. THP inhibited the complement activation in a dose‐dependent manner. BSA had no inhibition for the erythrocytes lysis. Data points are mean ± SEM of four repeated experiments. Inhibition ratio = [1 − (OD405 of serum with protein sample‐negative control)/(OD405 of 100% haemolysis‐negative control)] × 100 (Details is in method part)

Haemolytic assay was a typical functional study to test complement activity. Activation of each of the three complement pathways can induce haemolysis. We first set up a system to test the activation of lectin pathway, which was modified according to previous study.^21^ In mixture without THP, the haemolysis was nearly 100% and adding of THP could inhibit haemolysis of erythrocytes. Haemolysis inhibition ratio of THP was dose dependent when pre‐incubated THP and serum in 37°C. At the concentration of 10 μmol/L, THP could inhibit nearly 90% complement activity. When pre‐incubated BSA instead of THP, there was no inhibition of haemolysis (Figure 2).

**Figure 3:**
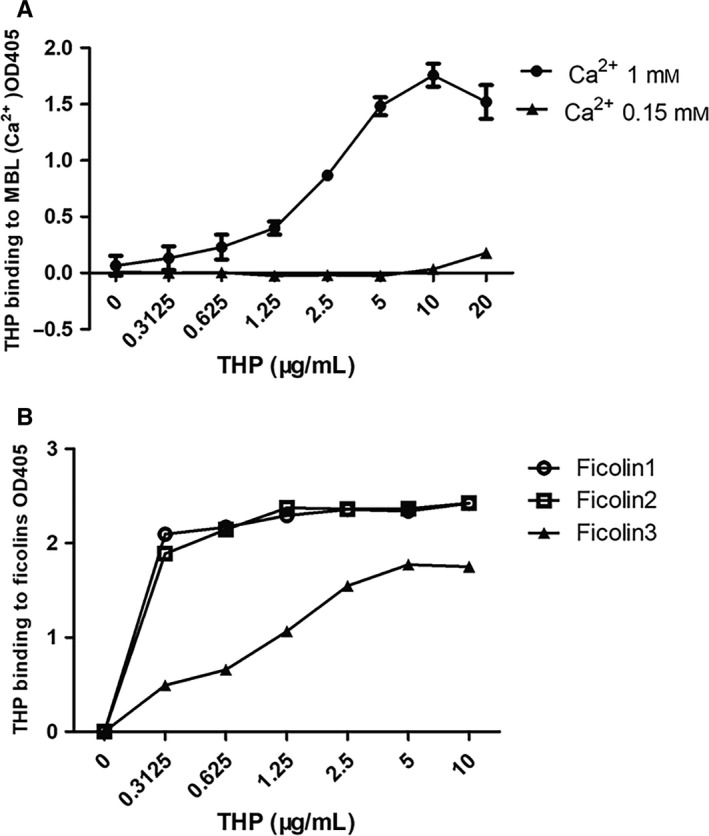
Binding of THP to MBL and ficolins. The binding of THP to MBL or ficolins was detected by ELISA, with fixed MBL and ficolin dose and different THP dosage. The experiment was performed in duplicate wells and repeated at three times. The binding of THP with other molecules was presented as value of OD405. Data points are mean ± SEM of three repeated experiments. A, The binding capacity of THP and MBL was dependent on Ca^2+^ concentration. B, The binding between THP with ficolin1 and ficolin 2 were stronger than ficolin

In order to identify which components in lectin pathway play crucial roles in haemolysis inhibition test. We also detected THP binding capacity with MBL and ficolins. The results showed that both MBL and ficolin‐1, 2, 3 could bind to THP in dose depended manner. But THP could not bind to MBL in VBS buffer with 0.15 mmol/L CaCl_2_. This result was supported by the previous evidence that MBL was a Ca^2+^ dependent protein.^26^ Binding capacity of THP with ficolin1 and ficolin2 was stronger than ficolin3 (Figure 3).

### 3.4 Change of N‐glycan spectra in patients with acute kidney injury, by MALDI‐TOF‐MS

**Figure 4:**
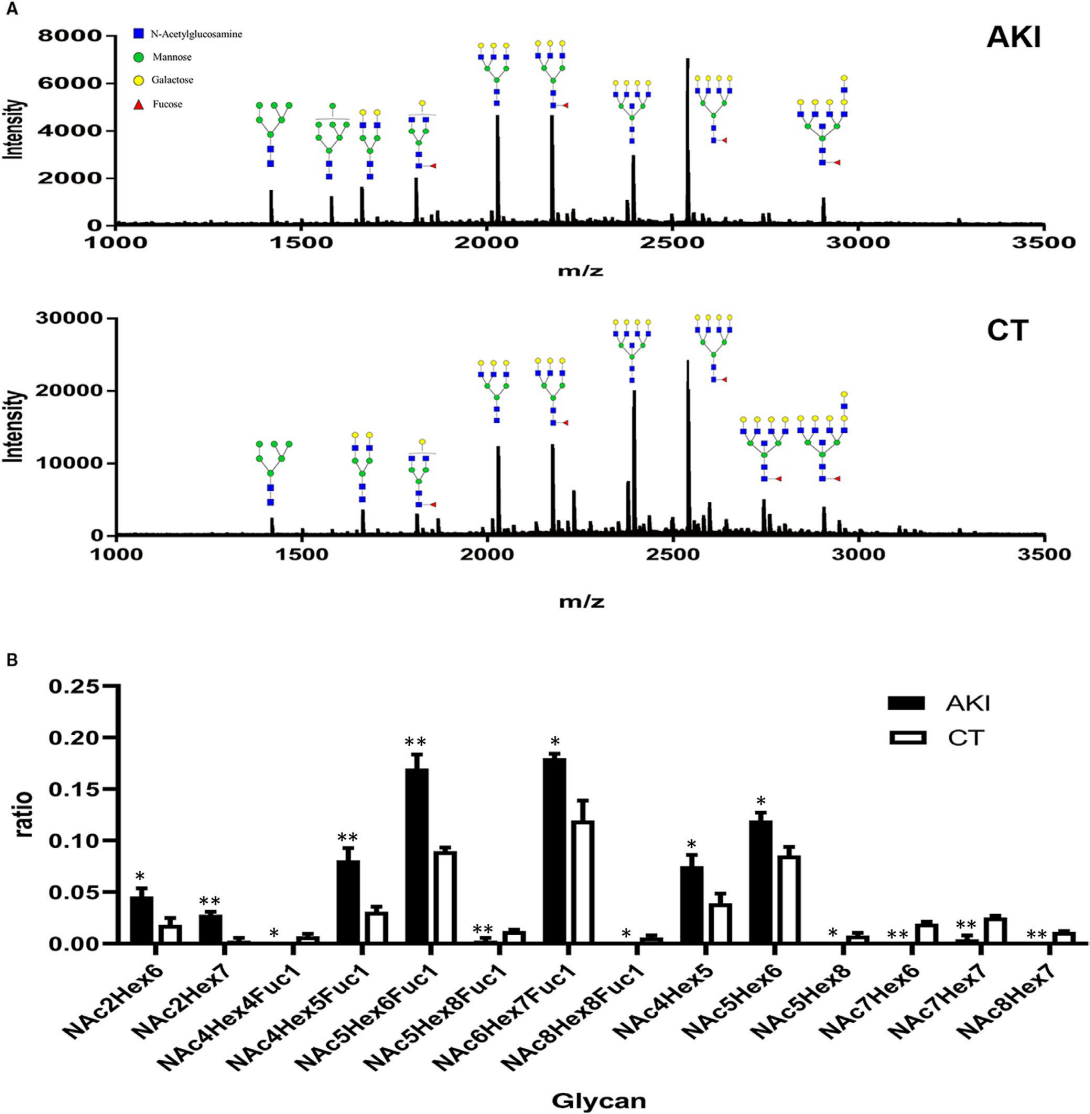
N‐glycosylation spectra of AKI patients and healthy controls by MALDI‐TOF‐MS. AKI: acute kidney injury, N = 10; CT: healthy control, N = 10. A, The typical N‐glycan profile released from uromodulin by MALDI‐TOF‐MS. Healthy control presented more complex N‐glycan structures. The amount of high‐mannose and core fucosylation increased in the AKI patients. B, Totally, 31 types of N‐glycan structures were found. The obvious different 14 glycans were showed in the figure. Raw data were normalized first, and independent sample *t* test was used to compare the difference between AKI patients and healthy controls. *P < .05, **P < .01. False discovery rate was used to correct the *P*‐values for multiple testing (Table S2)

Ten patients were confirmed with newly onset acute kidney injury after surgery (Table 1). The preliminary experiments showed that the sialic acids on THP interfered the detection of the N‐glycan profiles by MALDI‐TOF‐MS. Thus, we removed the sialic acids before lyophilized the glycans. Totally 31 types of N‐glycans were detected. Fourteen out of 31 types of N‐glycans were different in patients with AKI compared to control group. Significant differences were convinced by the FDR correction (Table S2). There are many diverse types and structural classes of N‐glycans that are detected by MALDI‐TOF‐MS and evaluation of structures can be done based on motif and context of the N‐linked glycan biosynthesis and processing pathways. Here, we presented the results of N‐glycan profile according to the high‐mannose, fucosylation and other complex N‐glycans. The normalized MS data showed that the glycans of high‐mannose and core fucosylation increased in AKI patients. Lower molecular weight di‐antennary complex N‐glycan also increased while heavier molecular glycans with poly‐antennary decreased in patients with AKI, compared to controls (Figure 4A,B).

### 3.5 Change of N‐glycans in patients with acute kidney disease, by lectin array

We also measured the N‐glycan changes using lectin array, as verification of MALDI‐TOF‐MS findings in 15 AKI patients (Table 1). Among these samples, five patients were measured with both two methods. Figure S2A displayed a representative picture of the profiling of lectin array and Figure S2B showed the lectin arrangement of the test chip. Statistical analysis indicated that 11 lectins (DBA, WFA, CNL, Lentil, Gal9, F17AG, GSL‐I, VFA, AAA, PHA‐P, BPA) presented significant differences (*P* < .05) between patients with AKI and healthy controls. The six lectins (DBA, WFA, CNL, Gal9, PHA‐P, BPA) bind with carbohydrates, which are added at later stage of glycosylation, decreased in AKI patients compared to controls. The less detection of these lectins indicated a lower level of complex glycans in AKI patients. Five lectins (Lentil, F17AG, GSL‐I, VFA, AAA) increased in patients with AKI compared to controls. Lentil binds to mannose or glucose and AAA recognizes fucose. Both of mannose and fucose are added to the THP at early stage of N‐glycosylation (Table 2). Other lectins recognizing fucose such as Lotus, RS‐Fuc, AAL had a trend of elevation in AKI although there were no significant differences (Table S1). There was no difference of sialylation in AKI patients and controls.

**Table 2:** Lectin array of AKI patients and healthy controls

Lectin name	Carbohydrate specificity	CT signal intensity	AKI signal intensity	Ratio_CT/AKI	*P* value
DBA	αGalNAc	5185.7 ± 716.9	2231.3 ± 521.7	2.324	.002
Gal9	poly LacNAc, GalNAcα1‐3Gal	350.5 ± 47.2	187.7 ± 29.4	1.868	.008
CNL	α/βGalNAc, GalNAcβ1‐4GlcNAc	94.9 ± 14.8	51.3 ± 6.2	1.849	.004
PHA‐P	(GlcNAcβ4Manα3)Manβ4,	1197.8 ± 246.3	1139.2 ± 194.7	1.578	.047
WFA	GalNAc	212.0 ± 24.0	137.2 ± 15.0	1.545	.002
BPA	Galβ3GalNAc	1197.4 ± 184.1	775.8 ± 107.6	1.544	.047
Lentil	d‐Mannose, d‐glucose	74.6 ± 2.0	93.3 ± 5.0	0.799	.004
F17AG	GlcNAc	74.4 ± 3.0	119.0 ± 11.4	0.625	.016
GSL‐I	αGal, α3GalNAc	82.7 ± 3.0	98.5 ± 5.7	0.840	.019
VFA	Poly β(1,4)GlcNAc	59.5 ± 2.3	68.2 ± 2.9	0.872	.040
AAA	αFuc	82.0 ± 2.3	95.7 ± 6.4	0.856	.040

Note

Statistic analysed by independent sample *t* test. Data were expressed as mean ± SEM.

Abbreviations: AAA, Anguilla anguilla; AKI, acute kidney injury, N = 15; BPA, Bauhinia purpurea; CNL, Clitocybe nebularis lectin; CT, healthy controls, N = 8; DBA, Dolichos biflorus; F17AG, *E coli* lectin; Gal9, Human galectin9 lectin; GSL‐I, Griffonia (Banderaea) simplicifolia I; Lentil, Lentil lectin; PHA‐P, Phaseolus vulgaris agglutinin; Ratio CT/AKI, signal intensity of control group over AKI group; VFA, Vicia faba; WFA, Wisteria floribunda.

### 3.6 Functional assays on the isolated THP from human AKI

**Figure 5:**
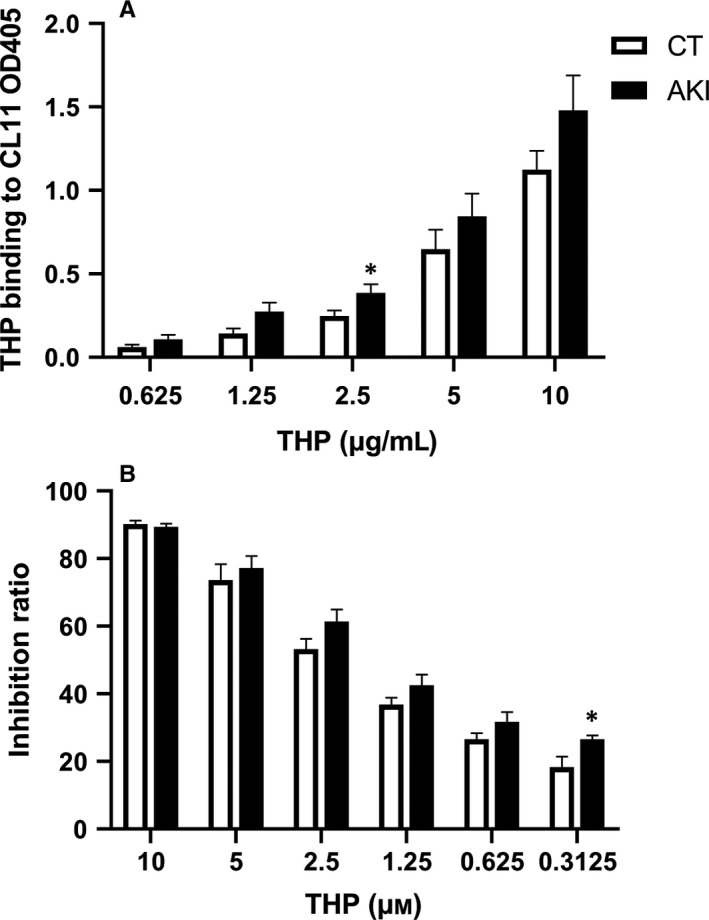
Comparison of the binding affinity and inhibition ability of THP isolated from healthy individuals and AKI patients. AKI: acute kidney injury; CT: healthy control. Independent sample *t* test was used to compare the difference. Data column was mean ± SEM. * *P* < .05. A, ELISA showed the binding affinity of CL‐11 and THP. The experiment was performed using THP isolated from nine AKI patients and nine healthy controls. Binding affinity of CL‐11 and THP at 2.5 µg/mL of THP reached significant p value (*P* = .037). B, THP inhibited complement lectin pathway activation detected by haemolysis assay. The experiment was performed using THP isolated from six AKI patients and six healthy controls. Inhibition ratio of THP at the concentration of 0.3125 µmol/L (0.3125 µmol/L = 27 µg/mL) reached significant *P* value (*P* = .03)

The binding of CL‐11 to THP isolated from AKI patients and healthy controls was also measured by ELISA. Binding strength of CL‐11 to THP collected from AKI patients was higher when compared to normal controls especially at 2.5 µg/mL of THP (Figure 5A). The haemolysis assay also showed that THP from AKI patients had enhanced inhibition on haemolysis of red blood cells, especially at the concentration of 0.3125 µmol/L (Figure 5B).

## 4 DISCUSSION

In this study, we identified a binding capacity between THP and CL‐11. The binding affinity was at 9.41 × 10^−7^ mol/L, lower than antigen‐antibody interaction,^27^ but similar with the binding affinity between THP and C1q or cFH.^7^, ^16^ The binding between THP and CL‐11 was in a dose‐dependent manner. By now, data from studies have revealed that THP can involve in all three complement activation pathways.^7^, ^16^ The binding between THP and C1q inhibited the activation of classical pathway, and binding between THP and cFH enhanced the degradation of C3b.^7^, ^16^ Both studies suggested that THP played roles in avoiding overactivity of classic and alternative complement pathway during injury. Thus, we further explored whether the binding between THP and lectin pathway initiation molecules influenced the complement activity with a modified haemolysis assay. In this assay, the effect of classical complement pathway was eliminated with anti‐C1q antibody.^21^ MBL is another important interference molecule need to be eliminated. Our data showed that high calcium concentration was required for the interaction between THP and MBL, but unnecessary for THP and CL‐11. We optimized the method by using MBL deficient serum and lowering calcium concentration to 0.15 mmol/L to ablate the binding of THP and MBL. The haemolysis assay results indicated that, the interaction between THP and CL‐11 inhibited the activation of lectin pathway. Although ficollins and other collectin molecules cannot be completely excluded from this system, this system partially proved that binding of THP and collectin could inhibit the activation of lectin pathway.

Collectin‐11 is a recently identified component of lectin pathway, synthesized by epithelial cells of the kidney.^12^ The CL‐11 contains four domains, N‐terminal domain, collagen‐like domain, neck domain and carbohydrate recognition domain (CRD).^28^ CL‐11 binds avidly to ligands possessing mannose or fucose,^15^ and the CRD is important for CL‐11 to recognize glycan ligands and glycoproteins.^29^ THP is a glycoprotein synthesized as a precursor of 640 amino acids.^30^ The predicted structure of uromodulin contains a leader peptide, four growth factor (EGF)‐like domains, a cysteine‐rich D8C domain, a zona pellucida (ZP) domain and a glycosylphosphatidylinositol‐anchoring site.^30^ The EGF‐like domains are likely important for protein–protein interactions while the ZP domain is important for THP polymerization 30 Glycosylation is an important post‐translational modification of THP. The carbohydrate moiety of THP is important for maintaining its structure and physiological function. The interaction between THP and cytokines (like IL‐1, TNF‐α) or complement C1q is based on the glycan structure.^31^, ^32^ CL‐11 appeared to direct the innate immune response against locally invasive factors^33^ following interaction with stress‐induced l‐fucose in renal epithelial cells.^13^ Thus, we used different enzymes to remove various glycans on the surface of THP. PNGaseF can remove all carbohydrate side chains without selection, but in non‐reduced condition, the 3‐dimensional structure of protein will influence the digestion of glycan chains. Fucosidase and EndoH are enzymes that can remove fucose and high‐mannose glycans specifically. In our study, binding between THP and CL‐11 was decreased with removal of different type of glycans, such as fucose, mannose or complex structure from THP. THP is a glycoprotein with core fucosylation.^30^ Removal of fucose with fucosidase completely ablated the binding between CL‐11 and THP, indicating that fucose on THP was crucial factor mediating the binding. Since the CRD region of CL‐11 was the major structure to recognize mannose or fucose, and the binding affinity was significantly decreased when removing the glycans from THP, we speculated that THP bound with CL‐11 via its CRD region.

Because the interaction of THP and CL‐11 is mediated by glycans on THP, it is interesting to know whether abnormal glycosylation exists during kidney injury. Mass Spectrometry and lectin array are common technologies suitable for glycosylation analysis. Mass spectrometry detects the intact glycosylation chains of glycoprotein while lectin is more specific at oligosaccharide. In this study, results of MALDI‐TOF MS and lectin array were basically consistent. Both of the two measurements showed an increase of mannose and fucose in AKI patients. MS also detected decreased complex structures, while lectin array analysis showed that GalNAc was the major declined complex structure in AKI patients. In this study, we found that glycans with high‐mannose or fucosylation were higher and glycans with more complex structure were lower in patients with AKI compare with controls. This phenomenon may be explained by the sequential glycosidase and glycosyltransferase reactions during N‐glycosylation. Usually the N‐glycan is first trimmed by glucosidases and mannosidases to Man5GlcNAc2, which serves as the precursor to further complicated glycan structures.^34^ Glycans that are not processed or incompletely processed by the mannosidases are named high‐mannose glycans. Fucosyltransferase eight catalyses the addition of α1,6‐linked fucose residues to the first GlcNAc residue, named as core fucosylation.^34^ High‐mannose glycans and fucosylation are early stage of glycosylation and usually with low‐molecular weight. More complicated poly‐antennary structures are generated based on early stage.^34^, ^35^ The more complex structure of glycans usually have high molecular weight. During tissue injury, the glycosylation procedure may be disrupted at different stage,^18^ thus the more complex structures tend to decrease. Similar phenomenon was also reported in another study.^36^ We then did a binding analysis and haemolytic analysis with THP from AKI patients to test whether abnormal glycosylation on THP form AKI patients can influence the interaction between the two proteins. Binding strength of CL‐11 with THP from AKI patients showed a trend of increasing, meanwhile, inhibition of lectin pathway activation tended to increase also. These results suggested the modification of glycosylation of THP may play a role in the pathogenesis of AKI.

Tamm‐Horsfall protein has been gradually recognized as a protective factor, as well as a biomarker of kidney injury. Many studies revealed that THP appeared to have anti‐inflammatory protective properties in kidney injury.^2^, ^7^, ^37^ Several small studies found that the excretion of THP was decreased during clinical AKI in the setting of cardiac surgery,^38^ intensive care unit 39and kidney transplant.^40^ And a study indicated that a higher uromodulin level in patients receiving a liver transplant associated with a lower risk of developing AKI.^41^ However, more studies are needed to determine whether a decreased urinary THP would be a good marker for AKI. THP is a glycoprotein rich of N‐glycans. The abnormal glycosylation of THP occurred even before the change of urinary protein level in disease status.^36^ It appeared that glycans mediated the immunoregulation functions of THP both in C1q binding activities^16^ as well as in this study. Glycosylation as an important post‐translational modification, may be sensitive to predict disease than protein level alone, like the disordered glycosylation of α‐fetoprotein could predict hepatocellular carcinoma.^42^ In this study, we demonstrated that THP can interact with CL‐11 and the interaction was mediated by glycans on THP, especially fucose. Meanwhile, abnormal glycosylation of THP exists in AKI patients, and the change tends to increase the interaction between THP and CL‐11. The results support the importance of THP glycosylation in AKI. CL‐11 could recognized fucose, fucosylated glycans and glycoprotein.^15^ L‐fucose accumulates at renal tubular cells after stress, recruits CL‐11 and then initiate lectin pathway.^13^ The exact sources of fucose on cell surface are not clear, the increased core fucosylation on THP may also be part of them, but more fucolysated THP may compete with other sources of fucose for binding with CL‐11 and inhibit overactivity of complements lectin pathway during renal injury. One of the functions of THP is associated with prevention of urinary infection, it is interesting to know whether THP change is related to sepsis‐induced AKI and AKI related infection. However, the aetiology of AKI patients was not uniformed, and it is hard to get a conclusion.

There are also limitations in our study. The sample size of the AKI cohort is small, due to difficulty of getting large dose of purified THP from AKI patients for MS measurement. Considering what we want to know is whether glycosylation change is a common phenomenon of AKI compared with normal status, we did not limit the type of surgery and selected controls with normal renal function without surgery. In the lectin microarray analysis, although only AAA reached significant p value, the other lectins also present the same trend. The limitation of sample size may be one of the reasons, except for tissue or protein specificity. Although the haemolysis assay was modified, influence of C1q and MBL were excluded, ficollins and other colletin molecules cannot be completely excluded from this system. However, the structure and carbohydrate ligand specificity of them are different. Ficolins recognize N‐acetylated molecule^43^ which is not obviously involved in AKI.

In summary, THP can bind to CL‐11, one of initiated molecules of lectin pathway and inhibit complement system activation via the lectin pathway. The interaction of THP and CL‐11 is mainly mediated by fucoses of THP. Higher level of fucose and mannose of THP could be found in acute kidney injury patients. We propose that this interaction between the lectin pathway and THP may provide a new insight for the immunomodulatory activities of THP.

## CONFLICT OF INTEREST

The authors confirm that there are no conflicts of interest.

## AUTHOR CONTRIBUTIONS

Yuqing Chen conceived and designed the study. Kunjing Gong, Min Xia and Yaqin Wang collected the samples and analysed the data. Kunjing Gong and Lufeng Bai performed the experiments. Wantao Ying provided MS analysis. Fengxue Zhu contributed to the sample collection. Kunjing Gong wrote the draft. Yuqing Chen, Fengxue Zhu and Wantao Ying reviewed and edit the manuscript. All authors approved the final version of the manuscript.

## Data Availability

The data that support the findings of this study are available from the corresponding author upon reasonable request.
