# Supplementary material for: Importance of glycosylation in the interaction of Tamm‐Horsfall protein with collectin‐11 and acute kidney injury
Source: J Cell Mol Med. 2020 Feb 11;24(6):3572–81. doi: 10.1111/jcmm.15046 (PMC7131921; doi:10.1111/jcmm.15046)
Supplement: Supplementary file 1 [file JCMM-24-3572-s001.pdf]

Importance of glycosylation in the interaction of Tamm-Horsfall protein with collectin-11 and acute kidney injury

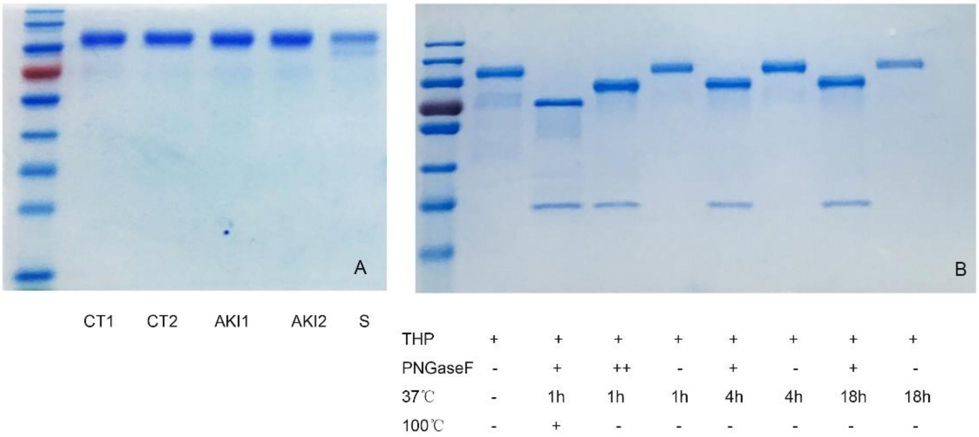

Supplementary figure1 Coomassie blue staining of THP samples

Coomasie staining of isolated THP and deglycosylated THP.

CT: healthy control; AKI: acute kidney injury; S: standard control

A. Lane1 and lane2 were samples from healthy control; lane3 and lane4 were samples from AKI patients; lane5 was a commercial product as standard control. All the purified samples were 2μg with similar molecular weight and band. The purity of THP was suitable for MS analyze.

B. lane1 was a native THP, lane 2 was denatured THP digested with PNGaseF at 37℃ for 1 hour. Lane 3 was native THP digested with a double dose of PNGaseF at 37℃ for 1 hour compare with native THP incubation at 37°C for 1 hour without digestion (lane 4). Lane 5 and 6 were 4 hours incubation. Lane 7 and 8 were 18 hours incubation. In the denatured condition, PNGaseF could remove all N-glycan from THP while part of the N-glycan could be removed from the THP in native condition. Extended incubation time did not contribute to a higher quantity of N-glycan release.

A)

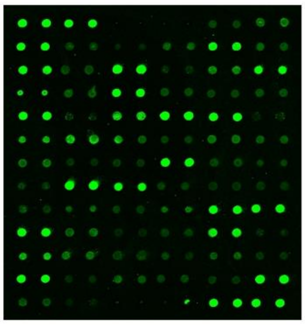

B)

	A B	C D	E F	G H	I J	K L
1	POS1	POS2	NEG	NEG	AAA	AAL
2	ACG	ACL	ASA	BanLec	BC2L-A	BC2LCN
3	BPA	Calsepa	CGL2	CNL	Con A	DBA
4	Discoidin I	Discoidin II	DSA	ECA	EEL	F17AG
5	Gal1	Gal1-S	Gal2	Gal3	Gal3C-S	Gal7-S
6	Gal9	GNA	GRFT	GS-I	GS-II	HHA
7	Jacalin	LBA	LcH	LEA	Lentil	Lotus
8	LSL-N	MAA	Malectin	MOA	MPL	NPA
9	Orysata	PA-IIL	PA-IL	PALa	PHA-E	PHA-L
10	PHA-P	PNA	PPL	PSA	PSL1a	PTL
11	RS-Fuc	SAMB	SBA	SJA	SNA-I	SNA-II
12	STL	UDA	UEA-I	UEA-II	VFA	VVA
13	WFA	WGA	NEG	NEG	POS2	POS1

Each lectin is spotted in duplicate horizonally

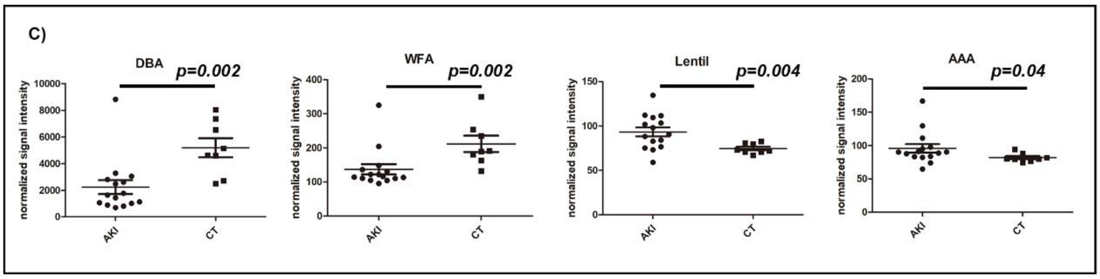

Supplementary figure 2. Change of glycans of AKI patients and healthy controls by lectin array.

AKI: acute kidney injury, N=15; CT: healthy control, N=8.

A. Typical profile of the lectin array, two of the adjacent were repetition.

B. The arrangement of the detectable lectins on the chip.

C. Four significant different lectins detected in the array analysis. DBA and WFA recognized GlaNAC, lentil recognized mannose and AAA recognized fucose. GlaNAC decreased in AKI patients, but mannose and fucose increased in AKI. Data points were mean±SEM

Supplementary table 1. Fucosylation detected by lectin-array

Lectin Name	Carbonhydrate specificity	CT signal intensity	AKI signal intensity	Ratio_ CT/AKI	P Value
**AAA**	αFuc	82.0±2.3	95.7±6.4	0.856	0.040
**AAL**	αFuc, Fucα6GlcNAc, SLe^x	217.5±13.8	247.4±13.7	0.879	0.141
**Lotus**	αFuc	58.0±2.8	67.5±3.6	0.859	0.115
**LCA**	αMan, αGlc, Fucα6GlcNAc	91.6±3.4	96.6±6.7	0.948	0.113
**RS-Fuc**	Fucose	364.8±30.0	427.2±40.5	0.854	0.114
**UEA-I**	αFuc	161.8±11.5	176.4±11.0	0.917	0.514

AKI: acute kidney injury, N=15; CT: healthy controls, N=8;
AAA: Anguilla anguilla; AAL: Aleuria aurantia; Lotus tetragonolobus; LCA: Lens Culinaris; RS-Fuc: Ralstonia solanacearum lectin; UEA-I: Ulex europaeus I.
Statistic analyzed by independent-sample t test. Data were expressed as mean±SEM

Supplementary table 2. Obvious different glycans detected by MALDI-TOF-MS

glycan	Ratio of AKI	Ratio of CT	P value	FDR
**NAc2Hex6**	0.0458±0.0078	0.0183±0.0066	0.021	0.015
**NAc2Hex7**	0.0280±0.0029	0.0028±0.0027	0.001	0.003
**NAc4Hex4F1**	0.00000000	0.0068±0.0025	0.009	0.010
**NAc4Hex5F1**	0.0807±0.0121	0.0309±0.0050	0.009	0.007
**NAc5Hex6F1**	0.1699±0.0137	0.0897±0.0034	0.001	0.003
**NAc5Hex8F1**	0.0027±0.0027	0.0122±0.0012	0.006	0.008
**NAc6Hex7F1**	0.1798±0.0045	0.1195±0.0193	0.023	0.015
**NAc8Hex8F1**	0.00000000	0.0059±0.0021	0.009	0.010
**NAc4Hex5**	0.0751±0.0109	0.0391±0.0095	0.048	0.026
**NAc5Hex6**	0.1193±0.0077	0.0856±0.0081	0.024	0.015
**NAc5Hex8**	0.00000000	0.0079±0.0026	0.009	0.010
**NAc7Hex6**	0.00000000	0.0195±0.0017	0.000	0.000
**NAc7Hex7**	0.0040±0.0040	0.0252±0.0020	0.003	0.005
**NAc8Hex7**	0.00000000	0.0113±0.0008	0.001	0.002

AKI: acute kidney injury, N=10; CT: healthy controls, N=10.

The ratio of glycan structure was normalized using flexAnalysis (Version 3.3). Data were expressed as mean±SEM.

Statistic analyzed by independent-sample t-test. The p-values were adjusted with FDR correction
